# Atezolizumab plus bevacizumab treatment for unresectable hepatocellular carcinoma: Early clinical experience

**DOI:** 10.1002/cnr2.1464

**Published:** 2021-06-11

**Authors:** Atsushi Hiraoka, Takashi Kumada, Toshifumi Tada, Masashi Hirooka, Kazuya Kariyama, Joji Tani, Masanori Atsukawa, Koichi Takaguchi, Ei Itobayashi, Shinya Fukunishi, Kunihiko Tsuji, Toru Ishikawa, Kazuto Tajiri, Hironori Ochi, Satoshi Yasuda, Hidenori Toyoda, Chikara Ogawa, Takashi Nishimura, Takeshi Hatanaka, Hideko Ohama, Kazuhiro Nouso, Asahiro Morishita, Akemi Tsutsui, Takuya Nagano, Norio Itokawa, Tomomi Okubo, Taeang Arai, Michitaka Imai, Yohei Koizumi, Shinichiro Nakamura, Kouji Joko, Hiroko Iijima, Yoichi Hiasa, Masatoshi Kudo, Real‐life Practice Experts for HCC (RELPEC) Study Group, and HCC 48 Group (Hepatocellular Carcinoma Experts from 48 Clinics in Japan)

**Affiliations:** ^1^ Gastroenterology Center Ehime Prefectural Central Hospital Kasuga‐cho Ehime Japan; ^2^ Department of Nursing Gifu Kyoritsu University Ogaki Japan; ^3^ Department of Internal Medicine Himeji Red Cross Hospital Hyogo Japan; ^4^ Department of Gastroenterology and Metabology Ehime University Graduate School of Medicine Ehime Japan; ^5^ Department of Gastroenterology Okayama City Hospital Okayama Japan; ^6^ Department of Gastroenterology and Hepatology Kagawa University Kagawa Japan; ^7^ Division of Gastroenterology and Hepatology, Department of Internal Medicine Nippon Medical School Tokyo Japan; ^8^ Department of Hepatology Kagawa Prefectural Central Hospital Takamatsu Japan; ^9^ Department of Gastroenterology Asahi General Hospital Asahi Japan; ^10^ Department of Gastroenterology Osaka Medical College Osaka Japan; ^11^ Center of Gastroenterology Teine Keijinkai Hospital Sapporo Japan; ^12^ Department of Gastroenterology Saiseikai Niigata Hospital Niigata Japan; ^13^ Department of Gastroenterology Toyama University Hospital Toyama Japan; ^14^ Hepato‐biliary Center Matsuyama Red Cross Hospital Matsuyama Japan; ^15^ Department of Gastroenterology and Hepatology Ogaki Municipal Hospital Gifu Japan; ^16^ Department of Gastroenterology Takamatsu Red Cross Hospital Takamatsu Japan; ^17^ Department of Internal Medicine, Division of Gastroenterology and Hepatology Hyogo College of Medicine Nishinomiya Japan; ^18^ Department of Gastroenterology and Hepatology Saiseikai Maebashi Hospital Maebashi Japan; ^19^ Department of Gastroenterology and Hepatology Kindai University Osaka Japan

**Keywords:** albumin‐bilirubin score, atezolizumab plus bevacizumab, hepatic function, lenvatinib, unrespectable hepatocellular carcinoma

## Abstract

**Background:**

Although atezolizumab plus bevacizumab (Atez/bev) treatment has been developed for unresectable hepatocellular carcinoma (u‐HCC), changes in hepatic function during therapy have yet to be reported.

**Aim:**

This retrospective clinical study aimed to elucidate early responses to Atez/Bev.

**Methods:**

From September 2020 to April 2021, 171 u‐HCC patients undergoing Atez/Bev treatment were enrolled (BCLC stage A:B:C:D = 5:68:96:2). Of those, 75 had no prior history of systemic treatment. Relative changes in hepatic function and therapeutic response were assessed using albumin‐bilirubin (ALBI) score and Response Evaluation Criteria in Solid Tumors (RECIST), ver. 1.1, respectively.

**Results:**

In initial imaging examination findings, objective response rates for early tumor shrinkage and disease control after 6 weeks (ORR‐6W/DCR‐6W) were 10.6%/79.6%. Similar response results were observed in patients with and without a past history of systemic treatment (ORR‐6W/DCR‐6W = 9.7%/77.8% and 12.2%/82.9%), as well as patients in whom Atez/Bev was used as post‐progression treatment following lenvatinib (ORR‐6W/DCR‐6W = 7.7%/79.5%), for which no known effective post‐progression treatment has been established. In 111 patients who underwent a 6‐week observation period, ALBI score was significantly worsened at 3 weeks after introducing Atez/Bev (−2.525 ± 0.419 vs −2.323 ± 0.445, *p* < .001), but then recovered at 6‐weeks (−2.403 ± 0.452) as compared to 3‐weeks (*p* = .001). During the observation period, the most common adverse events were appetite loss (all grades) (12.3%), general fatigue/hypertension (all grades) (11.1%, respectively), and urine protein (all grades) (10.5%).

**Conclusion:**

Atez/Bev might have therapeutic potential not only as first but also later‐line treatment of existing molecular target agents. In addition, this drug combination may have less influence on hepatic function during the early period, as the present patients showed a good initial therapeutic response.

## 1 INTRODUCTION

Hepatocellular carcinoma (HCC) is the most commonly encountered primary liver malignancy and sixth most common malignancy worldwide.^1^ In addition to curative treatments, such as liver transplantation,^2^ surgical resection,^3^ and radiofrequency ablation (RFA),^4^ and the palliative treatment transcatheter arterial chemoembolization (TACE),^5^ systemic therapeutic strategies have been developed for unresectable HCC (u‐HCC) cases.^6^, ^7^ Following introduction of sorafenib^8^ as the initial first‐line molecular target agent (MTA) in 2009, approval for lenvatinib^9^ as an additional first‐line MTA for u‐HCC was granted in 2018 in Japan. As for second‐line MTA treatments, regorafenib^10^ in 2017, ramucirumab^11^ in 2019, and cabozantinib^12^ in 2020 have received approval. This increase in therapeutic options has improved the prognosis of u‐HCC patients.^13^ Moreover, atezolizumab plus bevacizumab (Atez/Bev)^14^ recently developed in 2020 as a first‐line therapy using the combination of an immune‐checkpoint inhibitor (ICI) and MTA and is expected to show better therapeutic efficacy for improving prognosis of these patients than previously introduced first‐line MTA treatments (sorafenib, lenvatinib).^15^

Few reports regarding the influence of hepatic function during the early period of Atez/Bev therapy for u‐HCC are available at this time. Moreover, many u‐HCC patients continue to receive treatments with existing MTA drugs after approval of Atez/Bev, and there is scant information regarding the therapeutic efficacy of that combination given as later‐line therapy when treatment failure is observed in a patient receiving an MTA. In addition, there is no known effective post‐progression treatment for patients receiving lenvatinib.^16^

This study aimed to evaluate the influence of Atez/Bev on hepatic function as well as early therapeutic response when given not only as a first but also as a later‐line treatment in clinical practice.

## 2 MATERIALS AND METHODS

The present findings were obtained in a multicenter analysis of 171 u‐HCC patients treated with Atez/Bev from September 2020 to April 2021 at 16 different institutions. Therapeutic response was determined using Response Evaluation Criteria in Solid Tumors (RECIST), ver. 1.1.^17^ The first assessment of therapeutic effect was performed using dynamic CT results obtained at around 6 weeks after introduction of Atez/Bev, whenever possible, and additional dynamic CT examinations were performed as needed depending on the patient condition even before 6 weeks. Findings showing partial response (PR) at around 6 weeks were considered to indicate early tumor shrinkage for the present study (PR‐6W).

Patients with a known history of autoimmune disease were not treated with Atez/Bev. In addition, all patients were examined using upper gastrointestinal endoscopy for surveillance of esophageal and gastric varices. When detected or if a high risk of bleeding was present, the patient was treated according to local clinical practice.

### 2.1 Basal liver disease

For the present study, positive anti‐HCV findings were considered to indicate that HCC was due to hepatitis C virus (HCV), whereas HCC due to hepatitis B virus (HBV) was determined when the HBV surface antigen was positive. For patients with a history of alcohol abuse of 60 g/day or more,^18^, ^19^ basal liver disease was judged as alcoholic.

### 2.2 Liver function assessment

For assessment of hepatic reserve function, Child‐Pugh classification,^20^ albumin‐bilirubin (ALBI) grade,^21^, ^22^ and modified ALBI (mALBI) grade^23^ were utilized. ALBI grade 2 was divided into two subgrades (mALBI 2a and 2b) using an ALBI score of −2.27 as the cutoff value.^23^

### 2.3 HCC diagnosis and treatment

HCC diagnosis was based on an increasing course of alpha‐fetoprotein (AFP), as well as dynamic CT,^24^ MRI,^25^, ^26^ and/or pathological findings obtained during the clinical course. Barcelona Clinic Liver Cancer (BCLC)^27^ and tumor node metastasis (TNM) staging were determined based on criteria for TNM staging for HCC presented by the Liver Cancer Study Group of Japan (LCSGJ), sixth edition^28^ (TNM‐LCSGJ) and were used for evaluations of tumor progression.

### 2.4 Atez/Bev treatment and adverse event assessment

After obtaining written informed consent from the patient, intravenous Atez/Bev treatment, composed of 1200 mg of atezolizumab plus 15 mg/kg of body weight of bevacizumab, was given every 3 weeks. Treatment was discontinued following observation of any unacceptable or serious adverse event (AE) or clinical tumor progression. For assessment of AEs, the National Cancer Institute Common Terminology Criteria for Adverse Events, ver. 4.0,^29^ was used. The guidelines for Atez/Bev treatment provided by the manufacturer were used. At the time of Atez/Bev discontinuation, introduction of the next treatment was determined by the attending physician.

This study was conducted as a retrospective analysis of database records based on the Guidelines for Clinical Research issued by the Ministry of Health and Welfare of Japan after receiving official approval. All procedures were done in accordance with the Declaration of Helsinki. Written informed consent was received from all patients.

### 2.5 Statistical analysis

For statistical analysis, Welch's *t*‐test, Student's *t*‐test, Fischer's exac*t* test, a paired t test, Mann‐Whitney's *U* test, and the Friedman test were used as appropriate. For multiple comparisons, Bonferroni's method was utilized.

*p* values less than 0.05 were considered to indicate statistical significance. Easy R (EZR), ver. 1.53 (Saitama Medical Center, Jichi Medical University, Saitama, Japan),^30^ a graphical user interface for R (The R Foundation for Statistical Computing, Vienna, Austria), was used to perform all of the statistical analyses.

## 3 RESULTS

The median age of the patients was 73 years and 144 (84.2%) were male. One hundred sixty‐four (95.9%) were classified as Child‐Pugh class A. BCLC stage A was noted in 5, stage B in 68, stage C in 96, and stage D in 2, and 20 (11.7%) were classified as naïve HCC. Atez/Bev was given as the initial systemic treatment in 75 (43.9%) of the present patients, while sorafenib was used as previous treatments in 39, lenvatinib in 88, regorafenib in 17, ramucirumab in 9, and another ICI in 1 (including duplication). The median observation period was 2.25 months (Table 1). At the time of writing, Atez/Bev had been stopped in 29 (17.0%).

**TABLE 1:** Characteristics of patients treated with Atez/Bev (n = 171)

Age, yr^a^	73 (68‐80)
Gender, male: female	144:27
BMI, kg/m^2^ ^a^	22.9 (20.6‐24.9)
Etiology, HCV:HBV:alcohol:other	60:27:31:53
Positive for diabetes mellitus, %	63 (37.1%)
ECOG PS, 0:1:2	136:30:4:1
Platelets, ≥10^4^/μl^a^	13.4 (10.7‐17.3)
AST, U/L^a^	37 (28‐55)
ALT, U/L^a^	27 (19‐40)
T‐bilirubin, mg/dl^a^	0.70 (0.50‐0.92)
Albumin, g/dl^a^	3.8 (3.40‐4.10)
Prothrombin time, %^a^	91.5 (83.1‐101.9)
ALBI score^a^ (mALBI grade 1:2a:2b:3)	−2.520 (−2.207 to −2.720) (73:44:53:1)
Child‐Pugh class, A:B:C (Child‐Pug score, 5:6:7:8:9:10)	164:6:1 (112:52:5:1:0:1)
AFP, ng/ml	56.0 (6.8‐1029.2)
Tumor size, maximum, cm	3.0 (1.6‐6.0)
Intra‐hepatic tumors, n, none:single:multiple	15:18:138
MVI, Vp1:Vp2:Vp3:Vp4:Vv1:Vv2:Vv3,Vb2:Vb3	6:10:9:9:4:0:2:1:1
EHM, lung:bone:lymph node:perioneal:others	30:20:16:7:7
TNM‐LCSGJ, I:II:III:IVa:IVb	1:26:63:19:62
BCLC stage, A:B:C:D	5:68:96:2
Naïve HCC, %	20 (11.7%)
Initial dose of bevacizumab, mg^a^	880 (793.5‐1000.0)
Previous systemic therapies before Atez/Bev, n (none:1:2:3:4)	75:60:19:12:5
Past history of systemic therapy drug (SOR:LEN:REG:RAM:ICI)^b^	39:88:17:9:1
Infusion reaction, %	1 (0.6%) (grade 2)
Observation period, months	2.25 (0.82‐3.25)

Abbreviations: AFP, alpha‐fetoprotein; ALBI score, albumin‐bilirubin score; ALT, alanine aminotransferase; AST, aspartate transaminase; Atez/Bev, atezolizumab plus bevacizumab; BCLC stage, Barcelona Clinic Liver Cancer stage; BMI, body mass index; ECOG PS, Eastern Cooperative Oncology Group performance status; EHM, extra‐hepatic metastasis; HBV, hepatitis B virus; HCV, hepatitis C virus; ICI, immune‐check point inhibitor; LEN, lenvatinib; mALBI grade, modified ALBI grade; MVI, Macrovascular invasion; RAM, ramucirumab; REG, regorafenib; SOR, sorafenib; TNM LCSGJ sixth, tumor node metastasis stage by Liver Cancer Study Group of Japan sixth edition.

^a^ Median (interquartile range).

^b^ Duplication.

### 3.1 Initial evaluation of therapeutic response to Atez/Bev treatment

At the initial imaging evaluation performed at 6 weeks after starting treatment, complete response (CR) was not observed in 113 of the 171 patients, while at the time of writing PR as early tumor shrinkage has been noted in 12 (10.6%), stable disease (SD) in 78 (69.0%), and progression disease (PD) in 23 (20.4%) [objective response rate at 6 weeks (ORR‐6W), 10.6%; disease control rate at 6 weeks (DCR‐6W), 79.6%]. Patients with early tumor shrinkage (PR‐6W) showed higher rates of AE of hypertension (*p* = .032) as compared to the others (Table S1).

When Atez/Bev was given as first‐line treatment, PR‐6W (PR as early tumor shrinkage) was observed in 5 (12.2%), while SD was observed in 29 (70.7%) and PD in 7 (17.1%) (ORR 12.2%, DCR 82.9%). In cases that received Atez/Bev as later‐line treatment (ORR‐6W 9.7%, DCR‐6W 77.8%), PR‐6W was observed in 7 (9.7%), SD in 49 (68.1%), and PD in 16 (22.2%). In patients who had received lenvatinib and there were given Atez/Bev as post‐progression treatment (Table S2), PR‐6W was observed in 3 (7.7%), SD in 28 (71.8%), and PD in 8 (20.5%) (ORR‐6W 7.7%, DCR‐6W 79.5%) at the initial imaging evaluation (Table 2). From the view of etiology of HCC, initial therapeutic responses were similar in viral and nonviral patients (ORR‐6W/DCR‐6W: 10.8%/78.5% vs 10.4%/81.3%, *p* = .955).

**TABLE 2:** Initial response to Atez/Bev at 6 weeks, shown by RECIST ver. 1.1

	CR	PR	SD	PD	ORR‐6W/DCR‐6W	NE
All (n = 171)	None (0%)	12 (10.6%)	78 (69.0%)	23 (20.4%)	10.6%/79.6%	58
Atez/Bev as first‐line (n = 75)	None (0%)	5 (12.2%)	29 (70.7%)	7 (17.1%)	12.2%/82.9%	34
Atez/Bev as later‐line (n = 96)	None (0%)	7 (9.7%)	49 (68.1%)	16 (22.2%)	9.7%/77.8%	24
Atez/Bev as post‐progression treatment following LEN (n = 57)	None (0%)	3 (7.7%)	28 (71.8%)	8 (20.5%)	7.7%/79.5%	18

Abbreviations: Atez/Bev, atezolizumab plus bevacizumab; CR, complete response; DCR, disease control rate at 6 weeks; LEN, lenvatinib; NE, not examined at time of this analysis; ORR‐6W, objective response rate at 6 weeks; PD, progressive disease; PR, partial response; SD, stable disease.

### 3.2 Relative change in hepatic function in early period of Atez/Bev treatment

**FIGURE 1:**
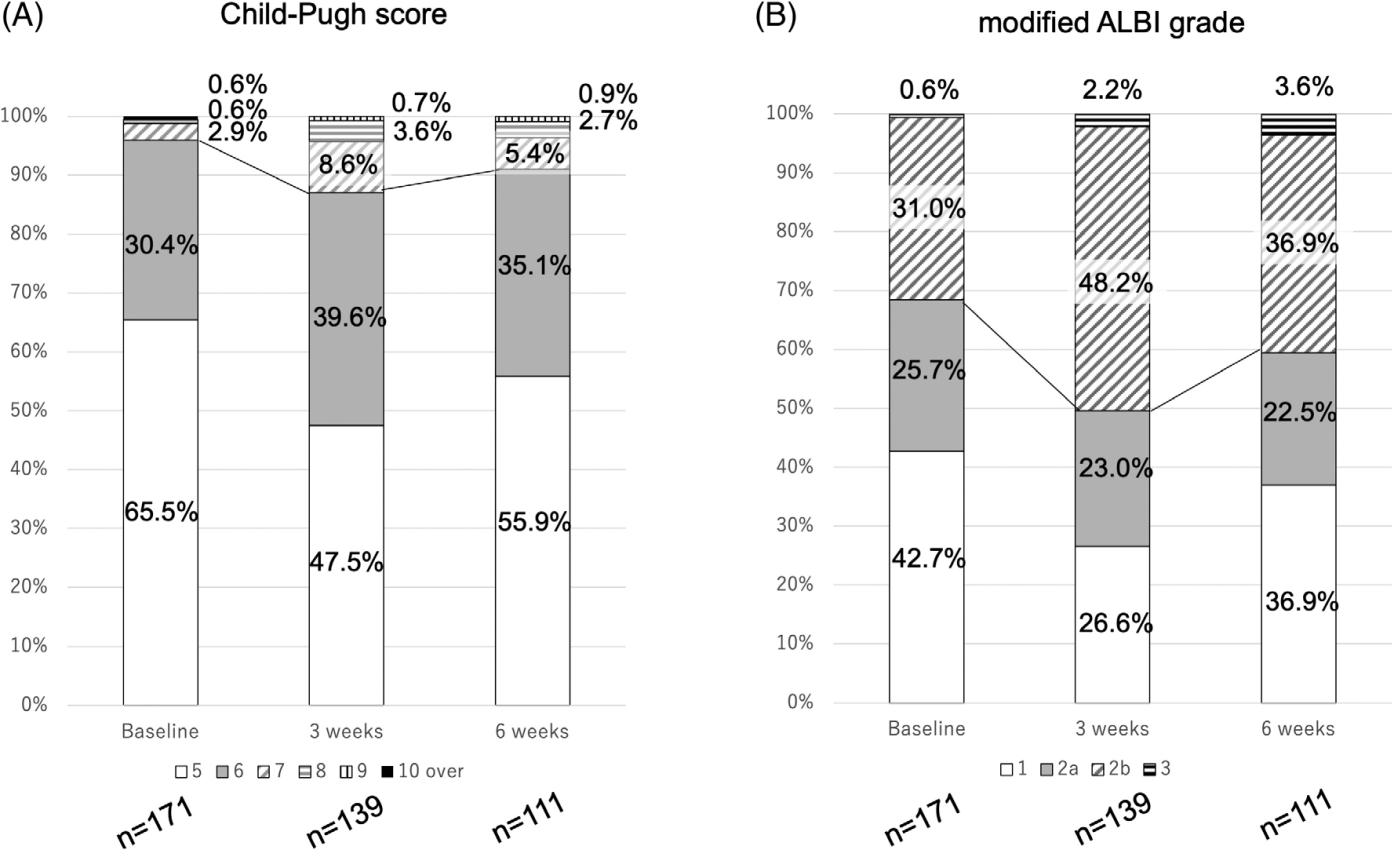
Relative changes in (A) Child‐Pugh score and (B) modified ALBI grade

**FIGURE 2:**
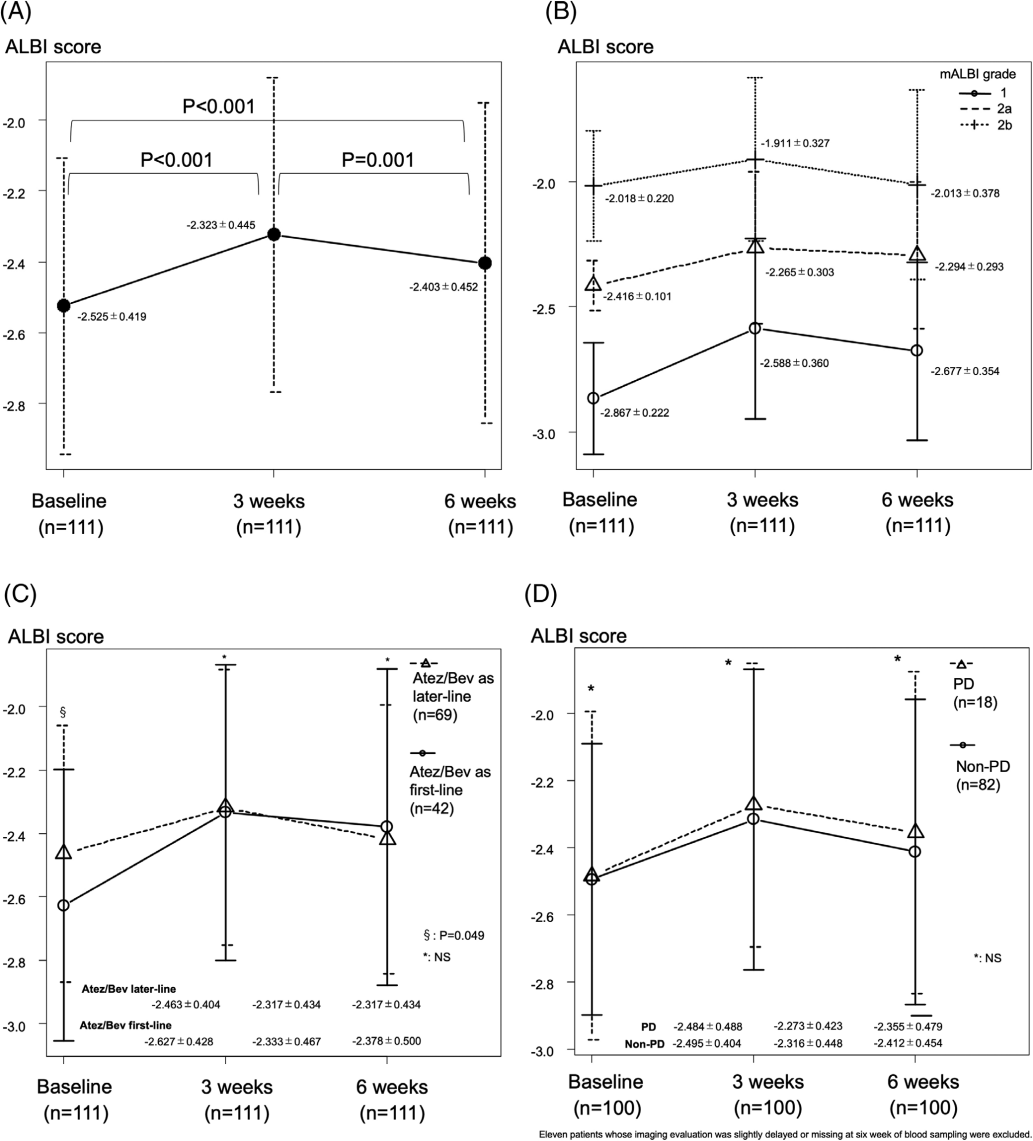
Patients who underwent observations after 6 weeks (n = 111). Relative changes in ALBI score for (A) all 111 patients (baseline, −2.525 ± 0.419; 3 weeks, −2.323 ± 0.445; 6 weeks, −2.403 ± 0.452), (B) 111 patients divided by mALBI grade, (C) 111 patients divided by therapy (Atez/Bev as first‐line vs Atez/Bev as later‐line), and (D) 100 patients divided by therapeutic response (non‐PD vs PD) after exclusion of eleven patients whose imaging evaluation was slightly delayed or missing at 6 week of blood sampling. NS, not significant

Child‐Pugh score and mALBI grade at baseline, as well as 3 and 6 weeks after starting Atez/Bev treatment, are shown in Figure 1. Child‐Pugh class A at those time points was 95.9%, 87.1%, and 91.0%, respectively (Figure 1A), while mALBI grade 1/2a was 42.7%/25.7% (68.4%), 26.6%/23.0% (49.6%), and 36.9%/22.5% (59.4%), respectively (Figure 1B). For the 111 patients who underwent the 6‐week examination , relative changes in ALBI score are shown in Figure 2. ALBI score deterioration was significant at 3 weeks after introducing Atez/Bev (−2.525 ± 0.419 vs ‐2.323 ± 0.445, *p* < .001). Although ALBI score at 6 weeks (−2.403 ± 0.452) was worse than that at the baseline (*p* < .001), it was improved as compared to that at 3 weeks (*p* = .001).

Similar relative changes of ALBI score were observed for each mALBI grade to that of the 111 patients who underwent the 6‐week examination (Figure 2B), as well as after dividing the patients into those who received Atez/Bev as initial systemic (n = 42) or as later‐line treatment at the baseline (n = 69) (−2.627 ± 0.428 vs −2.463 ± 0.404, *p* = .049), or at the three‐ (−2.333 ± 0.467 vs ‐2.317 ± 0.434, *p* = .851) or 6‐week (−2.378 ± 0.500 vs ‐2.417 ± 0.423, *p* = .670) time points (Figure 2C). In addition, there was no significant difference between non‐PD (n = 82) and PD (n = 18) cases at the baseline (−2.495 ± 0.404 vs ‐2.484 ± 0.488, *p* = .923), or at the three‐ (−2.316 ± 0.448 vs ‐2.273 ± 0.423, *p* = .702) or 6‐week (−2.412 ± 0.454 vs ‐2.355 ± 0.479, *p* = .649) time points (Figure 2D).

As for AEs, during the early period of Atez/Bev treatment, appetite loss (any grade) was most frequently observed (12.3%), followed by general fatigue/hypertension (any grade) (11.1%, respectively) and urine protein (any grade) (10.5%) (Table 3). At 3 and 6 weeks, treatment interruptions of Atez and Bev were 7.6% (10/132) and 11.4% (15/132), and 15.3% (17/111), and 16.2% (18/111), respectively.

**TABLE 3:** Adverse events with Atez/Bev in early period

	Any grade	Grade 1 or 2	Grade 3 or more
Appetite loss	21 (12.3%)	20 (11.7%)	1 (0.6%)
General fatigue	19 (11.1%)	18 (10.5%)	1 (0.6%)
Hypertension	19 (11.1%)	16 (9.3%)	3 (1.8%)
Urine protein	18 (10.5%)	6 (3.5%)	12 (7.0%)
Elevation of transaminase	12 (7.0%)	6 (3.5%)	6 (3.5%)
Fever	12 (7.0%)	11 (6.4%)	1 (0.6%)
Edema/ascites	12 (7.0%)	8 (4.6%)	4 (2.4%)
Thyroid function abnormality	9 (5.3%)	8 (4.7%)	1 (0.6%)
Rash	7 (4.2%)	5 (3.0%)	2 (1.2%)
Diarrhea/colitis	7 (4.2%)	4 (2.4%)	3 (1.8%)
Interstitial pneumonia	3 (1.8%)	1 (0.6%)	2 (1.2%)
Other AEs	42 (24.6%)	36 (21.0%)	6 (3.6%): EV rupture,^a^ nasal bleeding, pancreatitis, HCC rupture, acute heart failure, anemia (all 1)

Abbreviations: AE, adverse event; Atez/Bev, atezolizumab plus bevacizumab; HCC: hepatocellular carcinoma.

a Patients with Vp4.

## 4 DISCUSSION

The present results showed a DCR at the initial evaluation of therapeutic response performed at 6 weeks after beginning treatment with Atez/Bev similar to that seen in the IMbrave 150 trial,^14^ not only for all patients but also after dividing them into with and without past history of systemic treatments (MTA drugs). The IMbrave 150 trial (ASCO 2020)^31^ showed a time to response of 2.8 months (approximately at the time of the second imaging evaluation). At the time of writing, a high DCR‐6W and low early tumor shrinkage rate (ORR‐6W) were noted in the present cohort, though improved ORR following treatment is expected based on the results obtained after more 6 weeks.^31^ In these patients, AE of hypertension might be considered as a predictor of early tumor shrinkage, as well as hand‐foot skin reaction as seen with MTA treatments.^32^, ^33^ When PD was confirmed at the first imaging evaluation, overall survival (OS) was much worse than CR/PR and SD (8.0 months vs not estimated/16.1 months).^34^ Thus, the possibility of clinical efficacy with continued Atez/Bev treatment beyond PD should be considered. It will be necessary to establish a treatment strategy in the future that takes into account the initial PD result in patients undergoing Atez/Bev treatment.

Although it is well known that patients receiving sorafenib or lenvatinib show deterioration of hepatic function at 1 month after introducing treatment, both when given as first‐line^35^, ^36^ and as later‐line^37^ treatment, another study found no significant deterioration of hepatic function (ALBI score) in ramucirumab, anti‐VEGFR‐2, and placebo groups during each cycle of treatment.^38^ On the other hand, patients in the CheckMate‐459 trial who were treated with nivolumab showed small amounts of deterioration and recovery of ALBI score during the early treatment period.^39^ Although bevacizumab, an antibody that provides suppression upstream of the VEGFR‐2, is given as an anti‐VEGF‐A drug, Atez/Bev treatment might have a negative effect on hepatic function, such as seen with nivolumab, at least during the very early period of treatment. One of the reasons of the slight deterioration of the ALBI score at 3 weeks may be transient low grades of AEs of appetite loss and general fatigue at early period of Atez/Bev treatment. At 3 week, treatment interruptions of Atez and Bev were 7.6% and 11.4%, respectively, in the present cohort. For analyzing relationship between Atez/Bev and deterioration of hepatic function in detail, longer observation is necessary. However, it is possible that the present results show phenomena (deterioration and recovery of ALBI score) similar to those noted in the CheckMate‐459 trial.^39^

Better mALBI grade (1 or 2a) following drug introduction is an important factor in clinical settings for obtaining better therapeutic results with MTA treatments for u‐HCC patients^33^, ^37^, ^40^, ^41^, ^42^, ^43^ because a decline in hepatic function after introducing systemic therapy (sorafenib and lenvatinib) is commonly observed.^35^, ^36^ Ando et al. reported that better hepatic function (mALBI grade 1 and 2a) was the only significant predictive factor to indicate candidates suitable for post‐progression treatment after lenvatinib failure.^41^ Although a continuing deterioration of ALBI score was not observed in the present cohort, mALBI grade 2a might be the minimum hepatic function required for introducing Atez/Bev, even in Child‐Pugh class A patients as well those receiving MTA treatments, for expanding the clinical opportunity for introducing post‐progression sequential treatment. Although 56.1% of the present cohort were treated with Atez/Bev as later‐line treatment, our results indicated a therapeutic response similar to that seen in the IMbrave150 trial.^14^ Atez/Bev might also be a valid sequential therapeutic option for u‐HCC patients previously treated with MTA drugs including lenvatinib. Pianto et al. reported that worse ALBI grade (grades 2 and 3) (HR 2.1 and HR 3.1, *p* < .001, respectively), post‐ICI treatment (HR 0.30, *p* < .001), and non‐disease control (PD/non‐examination) (HR 4.88, *p* < .001) were significant prognostic factors related to OS in patients undergoing ICI treatment, while poor ALBI grade (grade 3) (HR 3.9, *p* < .001) and post ICI‐treatment (HR 0.3, *p* < .001) were significant prognostic factors also for OS post‐progression after ICI treatment.^44^ Introduction of systemic therapy, including Atez/Bev in u‐HCC patients with better hepatic function, if possible, should be kept in mind for increasing the opportunity for sequential treatment.^45^

Although initial therapeutic responses were similar in viral and nonviral patients in the present cohort (ORR‐6W/DCR‐6W: 10.8%/78.5% vs 10.4%/81.3%, *p* = .955), long‐term effect of Atez/Bev in each basal liver diseases [e.g. viral, nonviral, especially non‐alcoholic fatty liver disease (NAFLD)/non‐alcoholic steatohepatitis (NASH)] could not be analyzed at this writing. A recent report by Pfister et al. noted that therapeutic responses to ICI treatments differed according to the etiology of the basal liver disease.^46^ Meta‐analysis of the Pfister's report indicated that patients with HCC with a viral etiology showed therapeutic benefits from ICI use [HR 0.64], whereas those with a nonviral etiology did not [HR 0.92] (*p* = .03).^46^ Most importantly, results of two validation cohorts treated with ICI clearly showed that OS for NAFLD/NASH‐related HCC patients was significantly worse than that for the non‐NAFLD/NASH‐related HCC group (median OS: 5.4 vs 11.0 months, *p* = .023 and 8.8 vs 17.7 months, *p* = .034, respectively).^46^ Future analysis from this perspective is also necessary for Atez/Bev treatment.

Of course, existing first‐line treatments such as sorafenib and lenvatinib can continue to be the first choice for u‐HCC patients with autoimmune diseases. In any case, it is no doubt that better hepatic reserve function at the time of introducing first‐line treatments is important for improving the prognosis of u‐HCC in using any systemic treatments.

This study has some limitations. Although it was conducted as a multicenter study, the analysis was retrospective, and the observation period was short, making concrete conclusions not possible to obtain. Additionally, the therapeutic potential of Atez/Bev and determination of its influence on worsening hepatic function as compared to other existing MTA drugs, as well as administration in u‐HCC patients with mALBI grade 2b or 3 should be analyzed in the future. Accumulation of greater numbers of patients and a longer observation period will be needed for obtaining definitive conclusions.

In conclusion, Atez/Bev may have a good therapeutic potential when given not only as a first but also as a later‐line treatment following therapy with existing MTAs. This drug combination might have less influence on hepatic function during the early period. In early period examinations, the present patients showed a good initial therapeutic response.

## CONFLICT OF INTEREST

Atsushi Hiraoka, MD, PhD: lecture fees; Bayer, Eisai, Eli Lilly, Otsuka.

Takashi Kumada, MD, PhD: lecture fees; Eisai.

Masatoshi Kudo, MD, PhD: Advisory role: Eiasi, Ono, MSD, Bristol‐Myers Squibb, Roche. Lecture fees; Eisai, Bayer, MSD, Bristol‐Myers Squibb, Eli Lilly, EA Pharma. Research funding; Gilead Sciences, Taiho, Sumitomo Dainippon Pharma, Takeda, Otsuka, EA Pharma, Abbvie, Eisai.

The authors have stated explicitly that there are no conflicts of interest in connection with this article.

## AUTHOR CONTRIBUTIONS

**A.H.:** Conceptualization; data curation; formal analysis; project administration; writing‐original draft; writing‐review & editing. **T.K.:** Project administration; writing‐review & editing. **T.T.:** Data curation. **M.H.:** Data curation. **K.K.:** Data curation. **J.T.:** Data curation. **M.A.:** Data curation. **K.T.:** Data curation. **E.I.:** Data curation. **S.F.:** Data curation. **K.T.:** Data curation. **T.I.:** Data curation. **K.T.:** Data curation. **H.O.:** Data curation. **S.Y.:** Data curation. **H.T.:** Data curation. **C.O.:** Data curation. **T.N.:** Data curation. **T.H.:** Data curation. **H.O:** Data curation. **K.N.:** Data curation. **A.M.:** Data curation. **A.T.:** Data curation. **T.N.:** Data curation. **N.I.:** Data curation. **T.O.:** Data curation. **T.A.:** Data curation. **M.I.:** Data curation. **Y.K.:** Data curation. **S.N.:** Data curation. **K.J.:** Data curation. **H.I.:** Data curation. **Y.H.:** Data curation. **M.K.:** Writing‐review & editing.

## ETHICAL STATEMENT

The Institutional Ethics Committee of our hospital granted approval (IRB No. 30‐66). Written informed consent was received from all patients.

## Supporting information


**Table S1** Characteristics of patients with and without early tumor shrinkage (partial response) at 6 weeks.

**Table S2** Clinical features of patients treated with Atez/Bev post‐progression following lenvatinib (n = 57)

## Data Availability

The datasets generated during and/or analyzed during the current study are not publicly available. Due to the nature of this research, participants of this study did not agree for their data to be shared publicly, so supporting data is not available.
