# Supplementary material for: Atezolizumab plus bevacizumab treatment for unresectable hepatocellular carcinoma: Early clinical experience
Source: Cancer Rep (Hoboken). 2021 Jun 11;5(2):e1464. doi: 10.1002/cnr2.1464 (PMC8842687; doi:10.1002/cnr2.1464)
Supplement: Supplementary file 1 — Table S1 Characteristics of patients with and without early tumor shrinkage (partial response) at 6 weeks. [file CNR2-5-e1464-s002.docx]

Supplemental Table 1. Characteristics of patients with and without early tumor shrinkage (partial response) at 6 weeks.

|  | PR-6W (n=12) | Non-PR-6W (n=101) | P value |
| --- | --- | --- | --- |
| Age, years * | 75 (65-78) | 72 (67-80) | P=0.929 |
| Gender, male:female | 11:1 | 86:15 | P=1.0 |
| ALBI score *  (mALBI grade, 1:2a:2b:3) | -2.59 (-2.17 to -3.01)  6:2:4 | -2.44 (-2.19 to -2.74)  42:25:34 | P=0.473  (P=0.925) |
| Delta ALBI score at 3 weeks from baseline * | 0.18 (0.07 to 0.43) | 0.20 (0.04 to 0.34) | P=0.834 |
| Child-Pugh score, 5:6:7:8 | 7:5:0:0 | 63:34:3:1 | P=0.843 |
| Positive for diabetes mellitus, % | 2 (16.7%) | 37 (37.0%) | P=0.211 |
| AFP, ng/mL * | 279.2 (12.7-961.0) | 148.8 (8.6-2107.4) | P=0.929 |
| TNM-LCSGJ, I:II:II:IVa:IVb | 0:1:4:0:7 | 0:13:35:10:43 | P=0.769 |
| BCLC stage, A:B:C | 0:5:7 | 3:37:61 | P=0.833 |
| AE hypertension, Grade 0:1:2:3 | 8:2:2:0 | 89:1:9:2 | P=0.032 |

*Median (interquartile range). PR-6W: partial response at 6 weeks (early tumor shrinkage), ALBI score: albumin-bilirubin score, mALBI grade: modified ALBI grade, AFP: alpha-fetoprotein, TNM LCSGJ 6^th^: tumor node metastasis stage by Liver Cancer Study Group of Japan 6^th^ edition, BCLC stage: Barcelona Clinic Liver Cancer stage, AE: adverse event
