# Supplementary material for: Atezolizumab plus bevacizumab treatment for unresectable hepatocellular carcinoma: Early clinical experience
Source: Cancer Rep (Hoboken). 2021 Jun 11;5(2):e1464. doi: 10.1002/cnr2.1464 (PMC8842687; doi:10.1002/cnr2.1464)
Supplement: Supplementary file 2 — Table S2 Clinical features of patients treated with Atez/Bev post‐progression following lenvatinib (n = 57) [file CNR2-5-e1464-s001.docx]

Supplemental Table 2. Clinical features of patients treated with Atez/Bev post-progression following lenvatinib (n=57)

| Age, years * | 75 (70 to 80) |
| --- | --- |
| Gender, male:female | 51:6 |
| ECOG PS, 0:1 | 45:12 |
| Etiology, HCV:HBV:alcohol:other | 15:10:13:19 |
| Child-Pugh score, 5:6:7 | 39:17:1 |
| mALBI grade, 1:2a:2b | 22:17:18 |
| TNM LCSGJ, 1:2:3:4a:4b | 1:8:27:6:15 |
| BCLC stage, A:B:C | 3:29:25 |
| AFP, ng/mL * | 97.6 (16.5 to 1064.0) |
| Atez/Bev, second-:third-:fourth-:fifth-line | 43:8:4:2 |

* Median (interquartile range). Atez/Bev: atezolizumab plus bevacizumab, ECOG PS: Eastern Cooperative Oncology Group performance status, mALBI grade: modified albumin-bilirubin grade, TNM LCSGJ 6^th^: tumor node metastasis stage by Liver Cancer Study Group of Japan 6^th^ edition, BCLC stage: Barcelona Clinic Liver Cancer stage, AFP: alpha-fetoprotein
